# Apparent Effects of Opioid Use on Neural Responses to Reward in Chronic Pain

**DOI:** 10.1038/s41598-019-45961-y

**Published:** 2019-07-03

**Authors:** Katherine T. Martucci, Kelly H. MacNiven, Nicholas Borg, Brian Knutson, Sean C. Mackey

**Affiliations:** 1 Department of Anesthesiology, Perioperative and Pain Medicine, Division of Pain Medicine, Stanford University School of Medicine, Stanford, California, USA; 2 Department of Psychology, Symbiotic Project on Affective Neuroscience Laboratory, Stanford University, Stanford, California, USA; 3 Department of Anesthesiology, Duke University School of Medicine, Durham, North Carolina, USA

**Keywords:** Fibromyalgia, Reward

## Abstract

Neural responses to incentives are altered in chronic pain and by opioid use. To understand how opioid use modulates the neural response to reward/value in chronic pain, we compared brain functional magnetic resonance imaging (fMRI) responses to a monetary incentive delay (MID) task in patients with fibromyalgia taking opioids (N = 17), patients with fibromyalgia not taking opioids (N = 17), and healthy controls (N = 15). Both groups of patients with fibromyalgia taking and not taking opioids had similar levels of pain, psychological measures, and clinical symptoms. Neural responses in the nucleus accumbens to anticipated reward and non-loss outcomes did not differ from healthy controls in either fibromyalgia group. However, neural responses in the medial prefrontal cortex differed, such that patients with fibromyalgia not taking opioids demonstrated significantly altered responses to anticipated rewards and non-loss outcomes compared to healthy controls, but patients with fibromyalgia taking opioids did not. Despite limitations including the use of additional non-opioid medications by fibromyalgia patients taking opioids, these preliminary findings suggest relatively “normalized” neural responses to monetary incentives in chronic pain patients who take opioids versus those who do not.

## Introduction

Exogenous opioids are potent analgesics that exert their effects at the level of the spinal cord and by involving supraspinal sites via descending circuits (for review^1^). However, evidence suggests that long-term opioid use may not clinically improve the condition of individuals with chronic pain^2,3^. Current guidelines do not recommend opioids for the treatment of fibromyalgia, a condition of widespread chronic pain, due to the overlap between side effects of opioids (e.g., negative impacts on mood, sleep, cognition) and many co-occurring symptoms of fibromyalgia (e.g., impaired cognition, anxiety, depression, fatigue, poor sleep quality) as well as the potential for addictive behaviors when taking opioids^4^. Other than a small number of studies investigating efficacy of tramadol, there are no randomized controlled trials of pure mu-opioid agonists on fibromyalgia^5,6^. However, despite opioid treatment not proving superior to non-opioid treatment for chronic pain, some individuals with chronic pain report benefitting over 12 months when taking opioids, similar to their non-opioid taking counterparts^3^. Further, recent data from opioid taper clinical trials indicate that approximately 25% of patients with chronic pain taking opioids choose not to participate in opioid tapering (and almost another 25% withdraw), suggesting that these individuals may believe they are benefiting from opioids despite myriad known side-effects^7^. This discrepancy between clinical observations and patients’ beliefs is not trivial given significant and increasing concerns regarding the risks of opioid use, including overdose and addiction^8^. Prescription of opioids for the treatment of fibromyalgia, in particular, remains controversial and poorly informed.

While the use of long-term opioid medications might not be beneficial for chronic pain per se (i.e., in terms of not improving physical function and not reducing pain interference for example), it is possible that opioid medications could be benefiting brain reward processing and associated reward behavior in patients with chronic pain. Endogenous opioids are heavily involved in neural circuits implicated in reward processing^9^. Neural circuits implicated in reward processing include a network of cortical and subcortical regions including the nucleus accumbens (NAcc) within the ventral striatum, medial prefrontal cortex (MPFC), ventral tegmental area (VTA), amygdala, insular cortex, thalamus, and anterior cingulate cortex (ACC)^10,11^. Exogenous opioids can modulate reward-related behavior in animals (i.e., enhance drug reinforcement/place preference)^12^, and endogenous opioids partially mediate stress-induced analgesia in humans^13,12,13^. To date, a fair amount of published data exists on reward processing and effects of opioids on brain reward circuits in animal models of chronic pain^1,9^. In sharp contrast, limited data currently exist on how use of exogenous opioids influence reward processing in humans with chronic pain. It has been shown that individuals taking opioids (without chronic pain) have reduced functional connectivity (correlated activity) between the NAcc and other reward-related regions including the medial inferior orbitofrontal cortex^14^. Additionally, in individuals with chronic low back pain, rapid structural changes occur in brain regions involved in pain and reward processing (including the amygdala and orbitofrontal cortex) after only one month of opioid therapy^15,16^.

The study of reward and punishment is highly relevant for chronic pain. One of the most common features of someone in chronic pain, including fibromyalgia, is the development of fear-avoidance to movement, or kinesophobia^17^. This phenomenon is a great barrier to treating patients and helping improve their physical function and quality of life. Physical activity leads to pain (a punishment), which in turn progressively diminishes physical activity frequency, while avoidance of movement (and pain) is rewarding in the short-term which reinforces this maladaptive cycle. Thus, it is important to understand the mediators, moderators, and mechanisms of reward and punishment to develop and optimize safe and effective treatments to reduce or break this cycle. Chronic pain can alter neural function implicated in incentive processing, for example via reduced neurotransmission in motivational circuits in rodent models of chronic pain^18^ and as evidenced by reduced NAcc response to painful stimulus offset in chronic pain patients^19^. Further, individuals with chronic pain may have reduced brain opioid^20,21^ and dopamine^22^ function. Moreover, we have shown in a previous publication that individuals with fibromyalgia have reduced arousal to monetary incentive cues, reduced responses in the MPFC during reward anticipation, and increased MPFC response to non-loss outcomes (or avoidance of punishment)^23^.

Neural responses to rewards can be influenced by the experience of chronic pain, use of exogenous opioids, and potential for addictive behavior associated with opioid use - and all of these factors likely interact in complex ways. However, very limited data currently exist on how the use of exogenous opioids influences reward processing in humans with chronic pain.

To begin characterizing effects of opioid use on reward behavior and brain reward processing in patients with chronic pain, we studied a cohort of individuals with fibromyalgia taking opioids using a well-validated monetary incentive delay (MID) task^24^. The MID task manipulates potential monetary incentives involving gains (e.g., +$5 versus $0) and losses (e.g., −$5 versus $0) and yields measures of brain activity in response to both anticipation and outcome phases of these experiences across repeated trials. In healthy individuals participating in the MID task, reward anticipation typically elicits robust increases in bilateral NAcc activity, which correlates with ratings of positive aroused affect. This response is often accompanied by increased activity in the insular cortex (associated with risk), as well as other regions including the caudate and thalamus^25^. Conversely, reward outcomes typically elicit increased activity within regions of the MPFC, along with the caudate, amygdala and putamen, indicating that anticipation and outcome represent distinct components of reward processing^25^. In our previous publication, fibromyalgia patients not taking opioids show normal NAcc reward anticipatory activity, but reduced MPFC reward anticipatory activity, and increased MPFC response to non-loss outcomes (non-punishment), as compared with healthy controls^23^. In the present study, we compared a new cohort of fibromyalgia patients taking opioids to the previously published data set. While the effects of long-term opioid use on reward processing in chronic pain patients are generally unknown, we developed our current predictions based on these prior results. We specifically predicted that NAcc and MPFC responses to anticipated rewards and non-loss outcomes might be similarly altered in individuals with fibromyalgia taking opioids, as compared with healthy controls, and that this pattern would occur to a greater extent than in patients with fibromyalgia not taking opioids.

## Results

### Participants

Participants in the study underwent two sequential fMRI scans involving the monetary incentive delay (MID) task (MID-1 and MID-2, which used the same MID task but differently ordered trials) which together took approximately 20 minutes total. Post-scan cue-associated ratings of arousal and valence, and clinical and psychological questionnaires were collected to assess potential correlations between brain (fMRI) and behavioral data (see Methods for details). Seventeen healthy control participants, 18 individuals with fibromyalgia not taking opioids, and 19 individuals with fibromyalgia taking opioids signed informed consent for the study. Data from 5 participants were excluded because of an incomplete scanning session due to artifacts (N = 1 non-opioid fibromyalgia), excessive head motion (N = 2 controls, N = 1 opioid fibromyalgia), presentation of two MID-1 scans during the scans (different number of trials, and different trial order; N = 1 opioid fibromyalgia). Thus, data from 15 healthy controls and 34 females with fibromyalgia (17 taking opioids, 17 not taking opioids) were included in the analysis (Table 1).

**Table 1:** Participant Demographics.

	Controls	Non-Opioid	Opioid
Total Participants (all female)	15	17	17
Righthanded	14	16	17
**Self-Identified Race**			
Asian	6	2	0
African American	0	0	1
Caucasian	8	13	14
Other	1	2	2
Hispanic or Latina Ethnicity	1	3	2
**Employment Status**			
Part-time employed	2	3	2
Full-time employed	10	6	2
Unemployed	3	8	13
**Income Level**			
$0-$29,999	0	5	7
$30,000-$59,999	2	3	5
$60,000 or more	11	8	4
**Education Level**			
High School	0	3	0
College/University	7	11	11
Advanced Degree	8	3	6

“Non-opioid” and “Opioid” refer to the non-opioid fibromyalgia group and opioid fibromyalgia group respectively. One control participant did not indicate handedness, one non-opioid fibromyalgia subject was left handed. No participants were of race categories for Pacific Islander or Alaskan, or Native American (i.e., “other” refers to race other than all of these categories). High school refers to “up to or through high school”, college/university refers to “up to or through college/university”, and advanced degrees refer to “any amount of education post college/university”.

### Clinical, and psychological patient profiles

Responses to clinical and psychological questionnaires were collected from study participants (Table 2). The majority of these measures showed significant differences across the three groups due to differences between control and fibromyalgia groups. Post-hoc t-tests between the opioid and non-opioid fibromyalgia groups revealed similar levels of pain, psychological, and clinical measures. Additionally, no severe depression (i.e., no BDI scores greater than 30^26^) was observed in the fibromyalgia groups. Clinically significant anxiety (i.e., STAI-State scores greater than 40^27^) was observed in the non-opioid fibromyalgia group (N = 10) and opioid fibromyalgia group (N = 9).

**Table 2:** Clinical, Behavioral, and Psychological Measures.

	Controls		Non-opioid FM		Opioid FM		ANOVA	FM t-test
	N	Mean ± sd	N	Mean ± sd	N	Mean ± sd	P-Value	P-Value
Age	15	48.1 ± 10.2	17	48.1 ± 9.6	17	52.8 ± 6.9	0.237	0.295
Positive Affect (PANAS)	15	36.4 ± 5.4	17	26.4 ± 8.5	17	24.2 ± 7.2	p < 0.001	0.655
Negative Affect (PANAS)	15	13.1 ± 4.1	17	21.2 ± 8.0	17	21.6 ± 7.1	0.001	0.982
Behavioral Reward (BAS)	14	20.4 ± 2.9	16	20.4 ± 2.6	17	16.4 ± 8.1	0.412	0.453
Behavioral Drive (BAS)	14	13.7 ± 3.9	16	13.8 ± 3.6	17	9.3 ± 5.0	0.064	0.088
Behavioral Fun (BAS)	14	14.6 ± 2.3	16	13.3 ± 2.8	17	9.5 ± 5.0	0.034	0.147
Behavioral Inhibition (BIS)	14	21.1 ± 6.0	16	28.2 ± 3.5	17	23.1 ± 11.6	0.125	0.515
Mood Disturbance (POMS)	15	−4.5 ± 8.6	17	21.6 ± 15.8	17	24.2 ± 17.2	p < 0.001	0.862
Fatigue (PROMIS)	15	48 ± 6.5	17	65.5 ± 8.0	16	70.7 ± 5.0	p < 0.001	0.075
Trait Anxiety (STAI)	15	34.9 ± 8.3	17	49.7 ± 8.5	17	51.9 ± 12.4	p < 0.001	0.793
State Anxiety (STAI)	15	27.1 ± 7.5	17	41.4 ± 7.0	17	41.9 ± 12.8	p < 0.001	0.989
Depression (BDI)	15	2.3 ± 3.1	17	15.8 ± 8.9	17	15.2 ± 9.3	p < 0.001	0.973
Number of Pain Areas (FAF)	15	1.5 ± 2.0	17	13.9 ± 3.9	17	12.6 ± 3.7	p < 0.001	0.508
Pain Severity (BPI)	15	0.4 ± 1.0	17	5.7 ± 2.1	17	6.0 ± 1.5	p < 0.001	0.848
Pain Interference (BPI)	15	3.5 ± 6.4	17	36.9 ± 19.5	17	42.8 ± 17	p < 0.001	0.515

Participant counts for each measure differ from the total number of participants (controls N = 15, non-opioid fibromyalgia (FM) N = 17, opioid FM N = 17) because some participants did not complete all questionnaires. Abbreviations: PANAS, Positive and Negative Affect Schedule; BIS/BAS, Behavioral Inhibition System/Behavioral Activation System; PROMIS, Patient-Reported Outcomes Measurement Information System; STAI, State-Trait Anxiety Inventory; FAF, Fibromyalgia Assessment Form; BDI, Beck Depression Inventory; POMS, Profile of Mood States; BPI, Brief Pain Inventory; sd, standard deviation. Repeated measures analysis of variance (ANOVA) across the 3 groups (group effect) P-values are reported. Tukey post-hoc t-test values are reported for comparison between the two patient groups. Data are presented for descriptive purposes only, therefore significance values (P-Value) shown are not corrected for multiple comparisons.

Using a brief measure of fibromyalgia associated symptoms and severity (Fibromyalgia Assessment Form, based on the ACR Fibromyalgia Diagnostic Criteria^28^), both patient groups demonstrated similar number of painful regions across the body (Table 2). The number of painful areas ranged from 9–19 for non-opioid fibromyalgia participants and 6–19 for the opioid fibromyalgia participants out of a total 19 regions. The duration of pain symptoms reported ranged from 2–28 years with an average duration of 11.5 years (standard deviation of 7.7 years) in the non-opioid fibromyalgia group and 2–25 years with an average duration of 10.0 years (standard deviation of 7.0 years) in the opioid fibromyalgia group.

### Medication and opioid use

All patients were allowed to continue their usual medication use during the study. Medication, particularly opioid use, was recorded (by verbal report) from all study participants. In the opioid fibromyalgia group, the distributions of duration of opioid use and morphine equivalent daily dose were right-skewed with all individuals taking 1–75 mg except for one taking 355 mg, and all individuals taking opioids for 3 months −16 years except for one taking opioids for 40 years. Across all individuals with fibromyalgia taking opioids, the median duration of opioid use was 6.17 years and median morphine equivalent daily dose was 20 mg (N = 17). Excluding the one high opioid dose, the median morphine equivalent daily dose was 17.5 mg (N = 16). Excluding the one long duration opioid use, the median opioid use duration was 5.58 years (N = 16). Additional information on medications taken by all participants is provided in Table 3.

**Table 3:** Medications.

	Controls	FMN	FMO
Opioids	0	0	17
*Tramadol*			5
*Hydrocodone/acetominophen* (*Norco*)			9
*Morphine ER* (*MS-Contin*)			1
*Oxycodone/acetominophen* (*Percocet*)			2
*Codeine*			1
NSAID	0*	7	12
Acetaminophen	0	0	12
Topical Lidocaine Patch	0	1	0
SNRI (e.g., duloxetine)	0	4	7
SSRI (e.g., fluoxetine)	0*	2	4
Tricyclic Antidepressant (e.g., amitryptyline)	0	3	1
Other Anxiolytic (e.g., buspirone)	0	2	2
Antiepileptic (e.g., topiramate)	0	2	5
Triptans (e.g., sumatriptan)	0	0	6
Benzodiazepine	0	0	3
Benzodiazepine-like (e.g., eszopiclone)	0	0	2
Muscle Relaxant (e.g., cyclobenzaprine)	0	2	6
GABA Analogue (e.g., gabapentin)	0*	6	7
Low Dose Naltrexone	0	2	0
Medical Cannabis	0	1	0
SARI (e.g., trazodone)	0	0	4
NDRI (e.g., methylphenidate, buproprion)	0	0	3
Ondansetron	0	0	1
*Taking No Medications*	13	4	0

The number of individuals in each group taking the class of medications is shown for the control group, non-opioid taking fibromyalgia group (FMN) and opioid taking fibromyalgia group (FMO). *One control participant with premenstrual symptoms (2 days per month) reported taking gabapentin (100 mg/day, 2 days per month) and fluoxetine (40 mg/day), and another control participant reported taking celecoxib (200 mg) 3 weeks prior to the study visit due to a sports-related ankle injury. A post-hoc analysis excluding the 2 patients taking low-dose naltrexone did not change the results, therefore data from these patients were included in the presented analysis. Abbreviations: Nonsteroidal anti-inflammatory drug, NSAID; serotonin and noradrenergic reuptake inhibitor, SNRI; selective serotonin reuptake inhibitor, SSRI; gamma-aminobutyric acid, GABA; serotonin antagonist and reuptake inhibitor, SARI; norepinephrine-dopamine reuptake inhibitor, NDRI.

### Arousal and valence ratings

**Figure 1:**
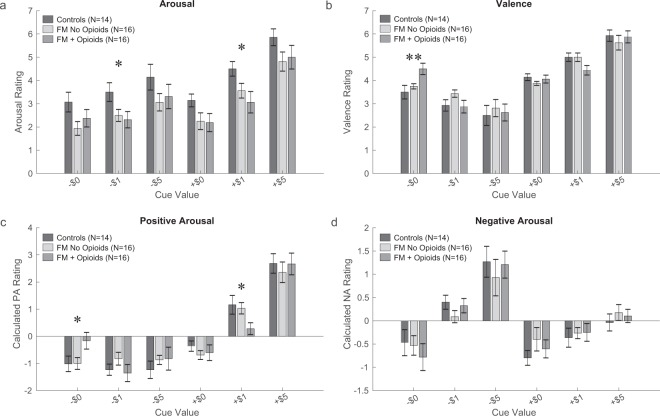
Behavioral Ratings. After the MID task fMRI experiment, study participants provided ratings of retrospective arousal and valence (7-point scale) to each of the 6 cues that had been presented during the MID task (e.g., +/− $0, $1, $5). Positive arousal and negative arousal ratings were calculated from ratings of arousal and valence. Two-way ANOVA was used for analysis of all behavioral values (effect of cue, effect of group, and cue by group interaction). (**a**) Arousal ratings showed a main effect of group [F(2,215) = 4.5; p = 0.017], main effect of cue [F(5,215) = 32.3; p < 0.001], and no group by cue interaction [F(10,215) = 0.3; p = 0.971]. (**b**) Valence ratings showed no effect of group [F(2,215) = 0.2; p = 0.840], a main effect of cue [F(5,215) = 61.3; p < 0.001], and no group by cue interaction [F(10,215) = 1.7; p = 0.088]. (**c**) Positive arousal showed a main effect of group [F(2,210) = 4.5; p = 0.017], main effect of cue [F(5,210) = 60; p < 0.001], and no group by cue interaction [F(10,210) = 1.3; p = 0.232]. (**d**) Negative arousal showed no effect of group [F(2,210) = 0.3; p = 0.739], a main effect of cue [F(5,210) = 20.8; p < 0.001], and no group by cue interaction [F(10,210) = 0.5; p = 0.918]. Asterisks indicate significant group differences for individual cues (*p < 0.05; **p < 0.01). One control and two fibromyalgia participants did not complete arousal and valence ratings after the scan.

After the MID task fMRI scans, separate ratings of arousal and valence were collected in response to each of the 6 MID task monetary cues presented (i.e., +$0, +$1, +$5, −$0, −$1, −$5). Both fibromyalgia groups had lower arousal ratings to all cues as compared with controls (group effect) [F(2,215) = 4.5; p = 0.017]. Additionally, arousal ratings varied based on cue type for all groups [F(5,215) = 32.3; p < 0.001]; however, there was no group by cue interaction [F(10,215) = 0.3; p = 0.971] (Fig. 1). Arousal ratings were not correlated with pain severity, duration of symptoms, anxiety, depression, or mood in the fibromyalgia groups, and none of these measures were significantly different between the opioid and non-opioid fibromyalgia groups.

Valence ratings varied based on cue type for all groups [F(5,215) = 61.3; p < 0.001]; however, there was no group effect [F(2,215) = 0.2; p = 0.840] and no group by cue interaction [F(10,215) = 1.7; p = 0.088] (Fig. 1). Positive arousal and negative arousal followed similar patterns as arousal and valence (see Fig. 1 for details).

### MID task reaction times and accuracy

Briefly, each trial of the MID task consisted sequentially of a cue presentation (potential gain cues: +$0, +$1, +$5; potential loss cues: −$0, −$1, −$5), short delay, target presentation, and outcome presentation. Participants needed to respond to the target (button press) as fast as possible to either gain money (for trials associated with gain cues) or avoid losing money (for trials associated with loss cues) in the previously shown cue amount (see Methods for details). To evaluate performance on the MID task among all groups, we analyzed reaction time and accuracy. Trials with more salient (i.e., higher) gain (+$5) and loss (−$5) cues produced faster reaction times as compared to trials with less salient cues (i.e., +$1, +$0, −$1, −$0) [F(5,225) = 9.2; p < 0.001] across all groups as expected. No group effect [F(2,225) = 0.5; p = 0.638] or group by trial (here, trial varies based on the different cues presented) interaction effect [F(10,225) = 1.2; p = 0.307] was observed for reaction time, indicating similar task performance (i.e., effort and speed) across all three groups.

This version of the MID task used an algorithm to target overall 66% accuracy rates across all trials; however, despite this automatic adjustment of task difficulty, trials with larger gain (+$5) and loss (−$5) cues resulted in higher accuracy as compared to trials with less salient cues (i.e., +$1, +$0, −$1, −$0) across all groups [F(5,230) = 4.8; p < 0.001]. No group effect for accuracy was observed [F(2,230) = 0.0; p = 0.981]. However, a group by trial (i.e., here, trials differ by cue type) interaction effect was observed for accuracy [F(10,230) = 2.1; p = 0.022]. Post-hoc within-group analyses revealed this interaction was due to significant effects of trial (i.e., here, trials differ by cue type) in the control group [F(5,70) = 3.3; p = 0.010] and in the non-opioid fibromyalgia group [F(5,80) = 3.5; p = 0.007], but not in the opioid fibromyalgia group [F(5,80) = 1.8; p = 0.114], which suggests that the opioid fibromyalgia group’s performance was less sensitive to trial (i.e., cue) type relative to the other groups.

### Region of interest: nucleus accumbens response

Brain reward anticipatory response was analyzed by comparing the blood oxygenation level dependent (BOLD) signal from preprocessed fMRI data during trials with +$5 cues versus trials with $0 cues for each participant. Thus, a gain versus no-gain anticipation (GVNant) task-based regressor was used to extract the BOLD signal at the relevant associated timepoints and average across multiple trials. The GVNant contrast was used to evaluate both NAcc and MPFC regions of interest (extracted beta values) for group differences in reward anticipatory response.

Brain reward outcome response was analyzed by comparing the BOLD signal from preprocessed fMRI data during successful (hit) trials with −$5 cues versus unsuccessful (miss) trials with −$5 cues for each participant. Thus, a non-loss versus loss outcome (NVLout) task-based regressor was used to extract the BOLD signal at the relevant associated timepoints and average across multiple trials. The NVLout contrast was used to evaluate both NAcc and MPFC regions of interest (extracted beta values) for group differences in non-loss outcome response. (See Methods and Supplementary Methods for details.)

**Figure 2:**
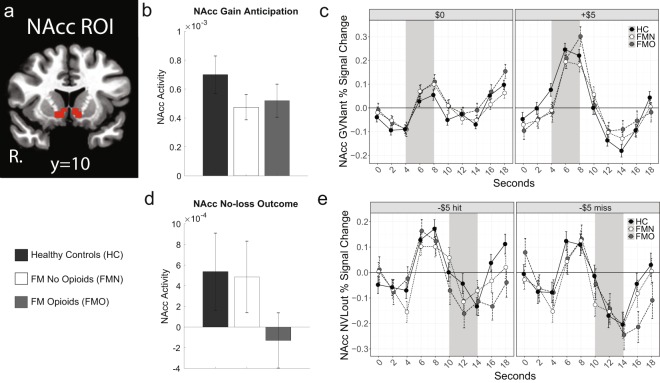
Nucleus Accumbens Activation during Gain Anticipation and No-loss Outcome. (**a**) Bilateral nucleus accumbens (NAcc) region of interest (ROI) is shown in red. (**b**) Extracted fMRI beta values from the NAcc ROI (“NAcc activity”) during gain anticipation (gain versus no-gain anticipation, GVNant contrast) were not significantly different among the three groups. (**c**) Raw time courses of NAcc activity [percent fMRI blood oxygenation level dependent (BOLD) signal change] for potential “no-gain” trials with $0 cues (left panel) and potential “gain” trials with +$5 cues (right panel). Gray shading indicates periods included (from which beta values were extracted for each condition) in each contrast. The anticipation fMRI BOLD response was estimated to correspond to 4–8 seconds [cue and fixation during TR 1 and TR 2 (0–4 seconds) plus a 4 second delay accounting for hemodynamic response function (HRF)]. (**d**) Extracted fMRI beta values from the NAcc ROI (“NAcc activity”) during no-loss outcome (no-loss versus loss outcome, NVLout contrast) were not significantly different among the three groups. (**e**) Raw time courses of NAcc activity [percent fMRI BOLD signal change] for “no-loss” [trials with −$5 cues and “hit” outcomes (i.e., net loss outcome equal to −$0; left panel)] and “loss” [trials with −$5 cues and “miss” outcomes (i.e., net loss outcome equal to −$5; right panel)]. Gray shading indicates periods included (from which beta values were extracted for each condition) in each contrast (“hit” minus “miss”). Outcome fMRI BOLD response was estimated to correspond to 10–14 seconds [cue and fixation during TR 4 and TR 5 (6–10 seconds) plus a 4 second delay accounting for HRF].

Due to its involvement as a key region implicated in reward processing, the bilateral NAcc was selected as a region of interest and beta values were calculated and extracted from each participants’ fMRI data using the GVNant and NVLout regressors. For reward anticipation (GVNant contrast), NAcc response was not significantly different across the three groups (one-way ANOVA) despite slight reductions in anticipatory response to monetary rewards in both patient groups compared to healthy controls [F(2,46) = 1.3, p = 0.286] (Fig. 2). Tukey post-hoc tests indicated no significant group differences between control and non-opioid fibromyalgia groups (p = 0.338), between control and opioid fibromyalgia groups (p = 0.357), nor between non-opioid fibromyalgia and opioid fibromyalgia groups (p = 0.999) for NAcc reward anticipatory response. Similarly, for non-loss outcome (NVLout contrast), NAcc response was not significantly different across the three groups (one-way ANOVA) [F(2,46) = 1.6, p = 0.211]. Tukey post-hoc tests indicated no significant group differences between control and non-opioid fibromyalgia groups (p = 0.993), between control and opioid fibromyalgia groups (p = 0.392), nor between non-opioid fibromyalgia and opioid fibromyalgia groups (p = 0.432) for NAcc non-loss outcome response.

### Region of interest: medial prefrontal cortex response

**Figure 3:**
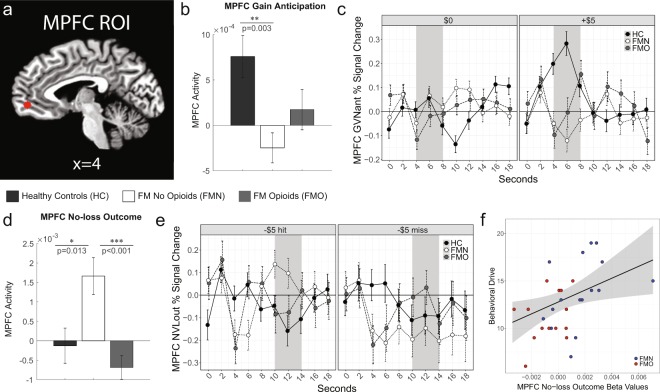
Medial Prefrontal Cortex Activation during Gain Anticipation and No-loss Outcome. (**a**) Bilateral medial prefrontal cortex (MPFC) region of interest (ROI) is shown in red (mirrored across cerebral hemispheres; but only one cerebral hemisphere is shown in this sagittal image). (**b**) Extracted fMRI beta values from the MPFC ROI (“MPFC activity”) during gain anticipation (gain versus no-gain anticipation, GVNant contrast) were significantly different among the groups, with post-hoc testing indicating this was driven by the difference between healthy control and non-opioid fibromyalgia (FM) groups. (**c**) Raw time courses of MPFC activity [percent fMRI blood oxygenation level dependent (BOLD) signal change] for potential “no-gain” trials with $0 cues (left panel) and potential “gain” trials with +$5 cues (right panel). Gray shading indicates periods included (from which beta values were extracted for each condition) in each contrast. The anticipation fMRI BOLD response was estimated to correspond to 4–8 seconds [cue and fixation during TR 1 and TR 2 (0–4 seconds) plus a 4 second delay accounting for hemodynamic response function (HRF)]. (**d**) Extracted fMRI beta values from the MPFC ROI (“MPFC activity”) during no-loss outcome (no-loss versus loss outcome, NVLout contrast) was significantly different among the three groups. (**e**) Raw time courses of MPFC activity [percent fMRI BOLD signal change] for “no-loss” [trials with −$5 cues and “hit” outcomes (i.e., net loss outcome equal to −$0; left panel)] and “loss” [trials with −$5 cues and “miss” outcomes (i.e., net loss outcome equal to −$5; right panel)]. Gray shading indicates periods included (from which beta values were extracted for each condition) in each contrast (“hit” minus “miss”). Outcome fMRI BOLD response was estimated to correspond to 10–14 seconds [cue and fixation during TR 4 and TR 5 (6–10 seconds) plus a 4 second delay accounting for HRF]. (**f**) Correlation between MPFC no-loss outcome fMRI beta values (NVLout contrast) and behavioral drive (BAS subscale) (r = 0.507, p = 0.004, N = 30). Four participants were missing BAS questionnaire data. Behavioral Activation Systems, BAS; medial prefrontal cortex, MPFC. ***p < 0.001, **p < 0.01, *p < 0.05.

Due to its involvement in reward processing, a bilateral MPFC region was selected as a volume of interest and beta values were calculated and extracted from each participants’ fMRI data using the GVNant and NVLout regressors. For reward anticipation (GVNant contrast), MPFC reward anticipatory response (one-way ANOVA across the 3 groups) revealed a significant effect of group in individuals with fibromyalgia compared to controls [F(2,46) = 6.1, p = 0.005]. Tukey post-hoc tests indicated significant group differences between control and non-opioid fibromyalgia groups (p = 0.003), but not between control and opioid fibromyalgia groups (p = 0.102), nor between non-opioid fibromyalgia and opioid fibromyalgia groups (p = 0.336). Thus, the overall group difference was driven by a significant reduction in MPFC reward anticipatory response in the non-opioid fibromyalgia group (Fig. 3B).

For reward outcome response (NVLout contrast), MPFC non-loss outcome response (one-way ANOVA across the 3 groups) revealed a significant effect of the fibromyalgia groups compared to controls [F(2,46) = 9.8, p < 0.001]. Tukey post-hoc tests indicated significant group differences between control and non-opioid fibromyalgia groups (p = 0.013) and between non-opioid fibromyalgia and opioid fibromyalgia groups (p < 0.001), but not between control and opioid fibromyalgia groups (p = 0.467) (Fig. 3D).

### Clinical, behavioral, and psychological correlations with region of interest fMRI results

To determine relationships between the fMRI results and clinical, behavioral and psychological measures, correlational analyses were conducted using extracted betas from the main contrasts [NAcc and MPFC reward anticipatory responses (GVNant betas) and non-loss outcome responses (NVLout betas)] and questionnaire scores for each participant. Across the patient groups, MPFC non-loss outcome responses (non-punishment, NVLout betas) were correlated with behavioral drive (BAS, behavioral activation system – drive subscale) (r = 0.507, p = 0.004, N = 30) (Fig. 3F). Additional trend-level correlations (that did not survive adjusted Bonferroni correction corrected threshold p < 0.007) included MPFC non-loss outcome response with BAS fun seeking subscale (p = 0.365, r = 0.047, N = 30) and MPFC reward anticipatory response (GVNant betas) with BAS reward responsiveness subscale (r = 0.397, p = 0.030, N = 30). No other ROI fMRI responses were correlated with questionnaire measures across the fibromyalgia groups (Supplementary Table S1).

### Impact of opioid duration, dose, and active phase

To further inform the findings of the fMRI and questionnaire analyses, additional analyses were performed within the opioid fibromyalgia group to determine potential effects due to specific characteristics of opioid use. Exploratory correlation analyses within the opioid fibromyalgia group identified that opioid dose (morphine equivalent) was correlated with positive affect (PANAS positive affect subscale, r = 0.483, p = 0.049, N = 17) and behavioral reward responsiveness (BAS reward responsiveness subscale, r = 0.547, p = 0.043, N = 14), however, after excluding the single fibromyalgia participant who was taking high dose opioids these relationships were no longer significant (PANAS positive affect subscale, r = 0.485, p = 0.057, N = 16; BAS reward responsiveness subscale, r = 0.169, p = 0.580, N = 13). No other relationships were identified for opioid dose, opioid use duration, ROI fMRI response (beta values), arousal, or other questionnaire variables.

Approximately half of the opioid fibromyalgia participants (N = 8 out of a total N = 17) were estimated (based on collected data: time of last dose and opioid medication type) to have undergone the fMRI scan during active phase of their opioid medication. Comparison of extracted ROI fMRI beta values revealed no significant differences between the two opioid sub-groups (active versus inactive opioid phases) for NAcc reward anticipatory response (GVNant betas) [t(14) = 0.8, p = 0.433], MPFC reward anticipatory response (GVNant betas) [t(14) = −0.6, p = 0.591] and MPFC no-loss outcome response (NVLout betas) [t(14) = −0.9, p = 0.389].

### Post-hoc whole brain ANOVA fMRI results

Post-hoc whole brain analyses were conducted to 1) confirm the ROI findings and 2) to identify additional brain regions that may be differently involved in reward processes among the groups. Whole brain fMRI data compared across the 3 groups for reward anticipation (GVNant contrast) and non-loss outcome (NVLout contrast) generally confirmed the observed group differences from the ROI analyses (Supplementary Results and Supplementary Figs S1 and S2). Whole brain results for LVNant and GVNout contrasts are presented in Supplementary Figs S3 and S4.

## Discussion

**Figure 4:**
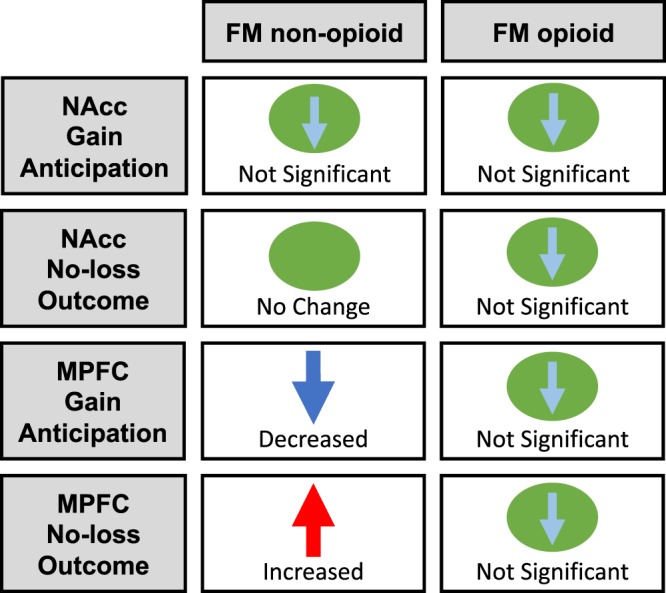
Summary of Main Findings. NAcc gain anticipation response was not significantly different from healthy controls in either non-opioid fibromyalgia (FM) and opioid fibromyalgia groups (gain versus no-gain anticipation, GVNant contrast) (top row). NAcc no-loss outcome response (non-punishment) was not significantly different from healthy controls in either non-opioid fibromyalgia and opioid fibromyalgia groups (no-loss versus loss outcome, NVLout contrast; second row). MPFC gain anticipation response was significantly decreased (blue arrow) in the non-opioid fibromyalgia group compared to healthy controls, but this decrease was not significant in the opioid fibromyalgia group (GVNant contrast; third row). MPFC no-loss outcome response was significantly increased (red arrow) in the non-opioid fibromyalgia group compared to healthy controls, and non-significantly decreased in the opioid fibromyalgia group (NVLout contrast; fourth row). (Light blue arrows within green circles indicate non-significant direction of response relative to controls; these are shown to inform future replication studies with larger sample sizes.) Fibromyalgia, FM; nucleus accumbens, NAcc; medial prefrontal cortex, MPFC.

In this sample of individuals with fibromyalgia taking opioid medications, we observed relatively normal brain activity in response to anticipation and outcome of monetary rewards and non-loss (relative to healthy controls) using fMRI during a monetary incentive delay task. This sharply contrasted with our previously published observations in a sample of individuals with fibromyalgia not taking opioids who demonstrated blunted brain response to anticipated rewards and enhanced response to non-loss outcomes (non-punishments) in the MPFC (see summary Fig. 4). Together, these findings indicate less altered brain response in individuals with fibromyalgia taking opioid medications during incentive processing, compared to individuals with fibromyalgia not taking opioids.

Across the non-opioid and opioid fibromyalgia groups, levels of pain, clinical symptoms, psychological measures, and behavioral measures were similar. The similarities between the two groups were incidental - in other words, we did not attempt to match fibromyalgia participants based on variables other than age (and sex). Therefore, among the cohorts that we had access to and recruited for the study, despite some participants taking opioids and others not taking opioids, the psychological and symptom profiles were comparable. This may have implications for reasonably high functioning (i.e., they were willing, able, and motivated to participate in the research study) people with fibromyalgia in the general population taking opioids at relatively low to moderate doses–in that they may generally have similar psychological, behavioral, and clinical profiles as compared with people of a similar status not taking opioids. Furthermore, although greater than the control group, relatively low levels of negative affect in our fibromyalgia groups (only 5 individuals with scores >30 and none >40 on the negative affect subscale, PANAS) may be related to lower pain scores and greater medication efficacy in our study population^29^. Similar levels of pain intensity, pain interference, and cognition have been identified between cohorts of individuals with chronic back pain taking (>3 months duration) and not taking opioid medications, despite lower self-efficacy beliefs and attention in the opioid group^30^. Currently, the number of neuroimaging studies comparing individuals with chronic pain who are taking versus not taking opioid medications are very limited. Further research is needed to provide increased understanding of the impact of opioid use on pain and psychological factors in chronic pain.

Despite the similar behavioral, psychological, and clinical profiles in the two fibromyalgia groups, we observed notable differences in the MPFC volume of interest response, specifically during gain anticipation and in response to non-loss outcomes. This indicates that functional neuroimaging of task-based reward processes may provide novel insights to the effects of opioids on brain reward systems (and behavior) in chronic pain that would otherwise not be identified from non-imaging sources such as behavioral testing or questionnaires.

We observed significant relationships between MPFC fMRI activity and behavioral drive, along with other trend relationships for additional BAS questionnaire subscale measures. Behavioral drive (and to a lesser extent, behavioral fun seeking) was positively correlated with MPFC non-loss outcome responses indicating that greater behavioral drive (and fun seeking) in the non-opioid fibromyalgia group may have contributed to heightened response to non-loss outcomes (i.e., non-punishment). Meanwhile, the opioid fibromyalgia group showed less behavioral drive related to less MPFC non-loss outcome response. From our uncorrected and exploratory analyses, behavioral reward responsiveness was correlated (uncorrected, p < 0.05) with MPFC gain anticipation response across the fibromyalgia groups and with opioid dose (morphine equivalent, uncorrected exploratory analyses p < 0.05) within the opioid fibromyalgia group. Thus, sensitivity to potential rewards appeared to be related to MPFC gain anticipation response and impacted by opioid use. Future analyses are needed, however, to confirm these exploratory and uncorrected findings.

The MPFC plays a critical role in processing of memory and emotionally salient stimuli and is widely interconnected with a network of regions related to these processes (for review^31^). The altered MPFC response in fibromyalgia may be due to reduced expectation of positive outcomes (during gain anticipation) and enhanced responses to non-losses due to its involvement in predicting reward probability^23,32^. Although unexpected, the findings of relatively normal MPFC activity (relative to controls) during gain anticipation and outcomes in the opioid fibromyalgia group suggests that these individuals may have higher expectations of positive outcomes (similar to controls, but in contrast to non-opioid fibromyalgia patients). If so, this could be due to the effects of opioid medication on brain reward systems or pre-existing factors–future investigations are needed to determine the relative contributions of actual opioid use/drug effects versus opioid preference/individual differences.

Multiple factors could influence the group differences in neural responses to incentives beyond those studied presently. Widespread pain is the predominant symptom of fibromyalgia, and although we measured the distribution, severity, and interference of pain symptoms across the fibromyalgia groups, these were not significantly different between fibromyalgia groups or correlated with neuroimaging findings. Cognitive symptoms are also present in fibromyalgia, but we did not directly assess cognition, which has been noted to be further impaired by opioids in chronic pain^33^. However, task performance (e.g., arousal, reaction time, and accuracy) was comparable between the fibromyalgia groups and controls, indicating minimal effects of cognitive impairments on the MID task. Additionally, fatigue was slightly greater in the opioid fibromyalgia group (assessed by Tukey post-hoc t-test, though not significant) and could have contributed to differences in neural responses to incentives.

The different neural responses between the non-opioid and opioid fibromyalgia groups could be due to several interacting factors across multiple psychological and neurophysiological domains. Expectations, shaped by both prior experiences and environment, can strongly contribute to different responses to medications and preferences for different types of medications. A randomized controlled trial of individuals with musculoskeletal pain to take opioids or not take opioids for 12-months identified that outcomes were similar across both groups, but expectations and experiences throughout the trial shaped patient responses to medications^34^. Thus, even the differences in neural responses to incentives observed across the opioid and non-opioid fibromyalgia groups could have resulted from different psychological, behavioral, and emotional networks formed prior to opioid use. However, the exploratory correlation between opioid dose and behavioral reward responsiveness suggests that the actual use of opioids in our cohort may contribute to the different neural responses to reward. Additional limitations include: 1) a lack of validation of opioid dose, 2) the use of more non-opioid medications in the opioid group (N.B. we have found it challenging to recruit sufficient fibromyalgia patients who were only taking opioids), 3) the possibility that the observed group differences might result from variability in fibromyalgia patients, and 4) modest sample size which limits our ability to identify associations of fMRI with behavioral data. Future studies investigating changes in neural responses to incentives may advance our understanding of how opioids influence human incentive processing and, in turn, the quality of life in chronic pain.

Together, these preliminary data suggest that neural responses to monetary incentives differ between individuals with fibromyalgia who are not taking versus taking opioid medications. The relatively “normalized” MPFC response to anticipated rewards and non-punishments in individuals with fibromyalgia taking opioids may relate to opioids’ ability to positively influence hedonic experience in chronic pain (i.e., or the belief of individuals with fibromyalgia that they benefit from opioid use), despite other potentially negative influences of long-term opioid use on chronic pain. Based on the current debate on the utility of opioids for long-term management of chronic pain, these findings may be controversial. We therefore encourage readers not to extrapolate these findings to suggest that opioids may, or may not, be of value in the treatment of fibromyalgia.

## Methods

### Participants

Thirty-seven females with fibromyalgia and 17 healthy females participated in the study. All fibromyalgia participants met the following inclusion criteria: American College of Rheumatology (ACR) 2011 criteria for fibromyalgia [(1) widespread pain index (WPI) score ≥ 7 + symptom severity (SS) score ≥ 5, or WPI score 3–6 + SS score ≥ 9, (2) similar level of symptoms present for at least 3 months, (3) no disorder to otherwise explain the pain], pain in 4 body quadrants, previous month average pain score of at least 2 (0–10 verbal scale), not pregnant or nursing, no MRI contraindications (e.g., claustrophobia, metal in body), and no uncontrolled depression or anxiety^28^. All fibromyalgia participants were allowed to continue their normal use of medications during the study. To be included in the non-opioid fibromyalgia group, individuals were not to be taking opioids as part of their treatment, not to have taken any opioids during the 90 days prior to study participation, and never to have taken opioids for a period greater than 30 days. To be included in the opioid fibromyalgia group, individuals were required to be have been taking opioid medications as part of their treatment for at least 3 months and at the time of study participation. Control participants were required to not have chronic pain, not be pregnant or nursing, have no MRI contraindications, and have no depression or anxiety.

### Study procedures

All procedures were approved by the Stanford University Institutional Review Board, were carried out in accordance with the approved protocols, and were conducted at the Stanford University Richard M. Lucas Center for Imaging. All participants signed written and informed consent acknowledging their willingness to participate in the study, understanding of all study procedures, and understanding that they were free to withdraw their study participation at any time. Data from the control and non-opioid fibromyalgia groups were analyzed previously and included in another publication^23^.

Prior to the scan session, study participants received instruction and practiced the MID task (for approximately 5 minutes) and use of arousal and valence rating scales. If necessary, the MID task practice was repeated until the participant understood the task and performed the task successfully. Participants underwent training on the rating scales for arousal and valence with written instructions presented on a laptop and explained to participants by trained personnel.

Questionnaires were administered to all subjects to determine clinical and psychological measures among the two fibromyalgia groups and control group. Questionnaires measured depression, anxiety, behavioral inhibition/approach, mood, positive and negative affect, pain distribution, pain intensity, pain interference, pain distribution, and fatigue (see Table 2 and Supplementary Methods for a list and references for questionnaires included in the analysis).

Structural MRI and fMRI data were acquired from all participants using a 3T General Electric scanner using an 8-channel head coil (GE Systems, Chicago, Illinois). The scan session consisted of a localizer scan, asset calibration scans, 2 MID task fMRI scans, and a T1 anatomical scan. The 2 MID fMRI task scans were acquired sequentially (no break in between) and with the following parameters: Gradient Echo Pulse Sequence with spiral in-out acquisition, flip angle of 76°, echo time (TE) of 30 seconds, repetition time (TR) of 2 seconds, sequential descending slice order, 32 oblique slices, slice thickness 4 mm, gap 0.5 mm, pixel size 3.43 mm. The spiral in-out scan sequence was used because it reduces orbitofrontal signal drop-out^35^ and improves acquisition of the medial prefrontal cortex and orbitofrontal cortex. The MID 1 fMRI scan included 266 volumes and the MID 2 fMRI scan included 302 volumes, excluding 12 s lead-in and 8 s lead-out. The T1 anatomical scan was acquired using 3D FSPGR (fast spoiled gradient-echo) IRprep BRAVO for registration of functional images and included whole brain, brainstem and cerebellum coverage; slice thickness 1 mm; frequency field of view (FOV) 22 mm, anterior/posterior frequency direction, 2 number of excitations (NEX), 11° flip angle, 6.8 TR, 2.6 TE, 256 frequency, 256 phase, 50.00 bandwidth.

### Monetary Incentive Delay (MID) fMRI Task

We ran the MID task in MATLAB (MATLAB R2012b, MathWorks, Natick, MA), using Psychophysics Toolbox for presentation of visual stimuli (Psychtoolbox-3^36^) via a screen placed above the participant in the scanner. Participant responses to the task were collected using a custom designed button box. A total of 90 trials were administered during the two MID task scans as performed previously^37^. Cues indicated the amount of money to be potentially gained (+$5.00, +$1.00, +$0.00) or lost (−$5.00, −$1.00, −$0.00) during each trial. After a 2 s delay (fixation cross) a target (triangle) was presented. Participants needed to initiate a response (button press) to either win (positive cues) or avoid losing (negative cues) the amount shown. Successful responses (i.e., occurring prior to target duration offset) generated a “hit” and unsuccessful responses generated a “miss”. Outcomes for each trial were presented as the amount gained (e.g., +$5.00), not gained (+$0.00), not lost (−$0.00), or lost (e.g., −$5.00). Target durations varied: initial target durations were set at the beginning of the scan (typically 250 ms) and programmatically adjusted throughout the task to target “hit” rate of approximately 66% across trials.

Arousal (low-medium-high) and valence (negative-neutral-positive) ratings were collected after the MID task fMRI scans using 7-point Likert scales. Participants were sent Amazon electronic gift cards for the combined total of gains and losses during the task after completion of the scan.

### fMRI Data preprocessing

Neuroimaging data were preprocessed using AFNI software (Analysis of Functional Neuroimages) [precompiled binary macosx_10.7_Intel_64: Jun 10 2016 (Version AFNI_16.1.21)] using custom scripts, and as performed previously^24^. First, 6 read-in and 9 read-out volumes were excluded from the scan data, the 2 MID fMRI scans were then concatenated into one series of images. Next, the functional images were slice time corrected, transformed from oblique to axial slice orientation, and aligned with the structural images. Motion correction was performed with 6 degrees of movement [translation (x, y, z), rotation (roll, pitch, yaw)]. The functional images were spatially smoothed using a 4 mm (full-width half-maximum, FWHM) Gaussian kernel^38^. Intensity normalization was applied by calculating the percent signal change per voxel and division by the average. We applied a 0.011 Hz high-pass filter to the functional data. Structural images were warped to Talairach space and functional images were warped to the Talairach warped structural images. A white matter (WM) and a cerebrospinal fluid (CSF) mask were created for each subject’s functional image based on signal intensity in seed loci in WM and CSF regions, respectively.

### fMRI Task contrasts

As used previously^24,37^ four separate orthogonal regressors contrasted responses to gain and loss during anticipation and outcome: gain versus no-gain anticipation (GVNant), loss versus no-loss anticipation (LVNant), gain versus no-gain outcome (GVNout), and no-loss versus loss outcome (NVLout). Contrasts compared gain and loss conditions to neutral (i.e., +/−$0) conditions, rather than to a no-stimulus baseline condition, in order to (1) focus on differences in reward/value processes in the brain and (2) to exclude general performance-related brain activity from the evaluated signal. Specifically, the contrasts included:

GVNant: anticipation during potential gain (+$5 cue) trials versus anticipation during potential no-gain (+/−$0 cue) trials; LVNant: anticipation during potential loss (−$5 cue) trials versus anticipation during potential no-loss (+/−$0 cue) trials; GVNout: hit (+$5 outcome) versus miss (+$0 outcome) during the outcome period for potential gain (+$5 cue) trials; NVLout: hit (−$0 outcome) versus miss (−$5 outcome) during the outcome period for potential loss (−$5 cue) trials.

The regressors of interest were convolved with a single gamma function approximating the hemodynamic response, with approximately a 6 second delay. We applied conservative motion censoring to the regressors so that any volume showing motion greater than 0.5 mm (and preceding volume) were excluded. Resulting activation maps “activity” for each participant represented blood oxygenation level dependent (BOLD) signal (a correlate of blood oxygenation levels which are a correlate of neural activity in fMRI) response corresponding to the regressor for each contrast separately. These individual activation maps were tested for group differences within *a priori* ROIs and post-hoc whole brain group comparisons. To maximize our chances of identifying significant group effects and consistent with previously published methods^23^, trials with +$1 cues were excluded from the fMRI contrasts because the strength of BOLD signal response for these trials was determined to be much lower than trials with +$5 cues (Supplementary Fig. S5).

### Region of Interest fMRI analyses

Region of interest (ROI) analyses focused on the NAcc and MPFC. We predicted that, in comparison to both control and non-opioid fibromyalgia, the opioid fibromyalgia group would show: 1) decreased NAcc activity during gain anticipation (GVNant) and no-loss outcome (NVLout), 2) decreased MPFC activity during gain anticipation (GVNant), and 3) increased MPFC activity during no-loss outcome (NVLout). We created the bilateral NAcc ROI by combining the left and right accumbens regions from the Desai Atlas (AFNI)^39^ and resampling to functional image resolution (52 voxels; voxel size = 2.9 mm × 2.9 mm × 2.9 mm). We created the bilateral MPFC ROI by drawing two conjoined 4 mm radius spheres (+/−4, 50, −3) within Brodmann Area 10 corresponding the frontal pole and resampling to functional image resolution (42 voxels). Additional information regarding the precise selection of the MPFC ROI can be found in the Supplementary Methods.

We extracted fMRI values from these ROIs and performed group statistical comparisons using analysis of variance (ANOVA) and post-hoc t-tests in SPSS (IBM SPSS Statistics for Macintosh, Version 22.0. Armonk, NY). Additional correlation analyses were performed between the ROI data and behavioral and clinical measures using SPSS.

### Clinical and psychological data analysis

Questionnaire data were quantified for each questionnaire separately and then assessed for group differences using ANOVA (SPSS). Post-hoc between group t-tests were then used to determine differences between individual groups (Tukey, SPSS). (See Table 2 and Supplementary Methods for list and descriptions of questionnaires).

### Behavioral analysis

We assessed performance and behavioral responses to the MID task by analyzing accuracy (percent hit rate), reaction times, and post-task ratings of arousal and valence to monetary cues. Additionally, measures of positive arousal and negative arousal were calculated from mean-deviated ratings of arousal and valence within subjects as done previously^24^. Each of these measures were assessed separately for differences across the 3 groups using a two-way ANOVA with factors of trial (here, “trial” simply refers to trials with different cues: +$0, +$1, +$5, −$0, −$1, −$5), group (HC, FMN, FMO), and trial by group interaction). Post-hoc one-way ANOVA tests were also used to determine group effects for each trial/cue separately (MATLAB, R2017a).

### MID Task fMRI analysis

Averaged beta values (parameter estimates) were extracted for each ROI from each participant for each contrast combination using custom scripts (MATLAB, AFNI). The extracted beta values were compared among groups using ANOVA (SPSS). We set the significance threshold to p < 0.0125 (initial p < 0.05 threshold Bonferroni corrected for 4 predetermined ROI x contrast comparisons: NAcc GVNant, NAcc NVLout, MPFC GVNant, MPFC NVLout). Additionally, raw time course data were plotted by extracting each participant’s fMRI data, sorting by trial type, and removing volumes with signal exceeding >4 standard deviations from mean activity^40^.

### fMRI Correlation analysis

Correlations between fMRI beta values (NAcc GVNant, NAcc NVLout, MPFC GVNant, and MPFC NVLout) and questionnaire variables were tested across the two fibromyalgia groups (2-tailed, bivariate, Pearson correlation, SPSS) (see Supplementary Methods for list of included questionnaires). Correlations between ROI fMRI beta values and questionnaire variables were corrected for multiple comparisons based on the inclusion of 7 independent (not correlated) measures (Supplementary Methods). Additional analyses were conducted to rule out potential influences of motion on the results and to confirm ROI findings with whole brain analyses (Supplementary Methods).

## Supplementary information

Supplementary Materials for Apparent Effects of Opioid Use on Neural Responses to Reward in Chronic Pain

## Data Availability

The datasets generated during and/or analyzed during the current study are available from the corresponding author on reasonable request.
