# Supplementary material for: Apparent Effects of Opioid Use on Neural Responses to Reward in Chronic Pain
Source: Sci Rep. 2019 Jul 3;9:9633. doi: 10.1038/s41598-019-45961-y (PMC6610070; doi:10.1038/s41598-019-45961-y)
Supplement: Supplementary file 1 — Supplementary Materials for Apparent Effects of Opioid Use on Neural Responses to Reward in Chronic Pain [file 41598_2019_45961_MOESM1_ESM.pdf]

Supplementary Materials for:

Apparent Effects of Opioid Use on Neural Responses to Reward in Chronic Pain

Katherine T. Martucci PhD, Kelly H. MacNiven PhD, Nicholas Borg MS, Brian Knutson PhD, Sean Mackey MD PhD

Supplementary Methods

Details of Questionnaires

Questionnaire measures included the following: The Behavioral Inhibition Systems / Behavioral Activation Systems (BIS/BAS)[1] measures avoidance/approach behaviors and negative affect/positive affect with the inhibition and activation subscales, respectively. The BAS portion of the questionnaire is divided into subscales of Behavioral Reward Responsiveness, Behavioral Drive, and Behavioral Fun Seeking. The Positive Affect and Negative Affect Scale (PANAS)[2] is a widely-used measure of positive and negative emotion assessed for the present or over the past week (at the discretion of the participant). The State Trait Anxiety Inventory (STAI)[3] separately measures "state" (transient; "how you feel right now") anxiety and "trait" (more stable, less transient ; "indicate how you generally feel") anxiety via its two subscales. The Profile of Mood States (POMS)[4] measures 6 dimensions of present mood which include tension/anxiety, anger/hostility, vigor/activity, fatigue/inertia, depression/dejection, confusion/bewilderment. Beck's Depression Inventory (BDI) [5] assesses depression "during the past two weeks, including today" and provides scores for a range of depression levels from normal "ups and downs" (scores 0-10) to extreme depression (scores 40-63). The Brief Pain Inventory (BPI) [6] (Short Form) is a chronic pain questionnaire that provides summed measures of pain severity and pain interference with general activity, mood, walking ability, normal work, relations with people, sleep, and enjoyment of life. The PROMIS (Patient Reported Outcome Measures Information Systems) Fatigue Scale measures the experience (frequency, duration, intensity) of fatigue and its impact on physical, mental, and social activity over the past 7 days,

and we used the computerized adaptive test (CAT) version 1[7]. The Fibromyalgia Assessment Form includes 19 body locations that can be denoted as painful and these are derived from the American College of Rheumatology revised 2011 criteria for fibromyalgia [8].

Note: The original BIS/BAS questionnaire by Carver and White uses a 4-point response scale (1 = strong agreement, 4 = strong disagreement) with no option for a neutral response [1]. Our BIS/BAS questionnaire unintentionally used a 5-point response scale which included a neutral response option. Thus, our BIS/BAS measurements may have been influenced by central tendency effects (i.e., preferential selection of the neutral response option).

Regarding Power for MID Task fMRI Analysis

Peak MID task fMRI NAcc reward anticipatory activation (+$5 versus +$0) results in the large reported effect size of $f^2 = 3.07$, and at least 6 participants are required to detect group effects with power equal to .80 ($p < 0.05$) [9,10]. Previous investigations support between-group differences for NAcc MID task fMRI data with sample sizes of 12 to 19 per group [11,12]. Therefore, we estimate that our group sample sizes should be large enough to detect group differences in fMRI activation in response to the MID task design. While our sample sizes are modest, we believe that they are sufficient to provide meaningful results for the generation of data to support future hypotheses and subsequent/replication investigations. We also note that reasonably powered comparisons have been made in schizophrenia using the MID task (group sizes of: 22 patients with schizophrenia, 24 with patients with depression, and 21 controls), so we believe it is reasonable to generate similar hypotheses with our somewhat smaller sample [13,14].

Region of Interest Additional Details

The NAcc and MPFC regions of interest (ROI) used in the present analysis were identical to those used in our previous publication [15]. Specifically, for the MPFC ROI selection, we created a

MPFC ROI as two conjoined spheres (4mm radius) at +/-4, 50, -3 (Brodmann Area 10 / frontal pole). We chose these coordinates primarily from a previous study using the MID task and analyzing a MPFC ROI in healthy controls and major depressive disorder patients (MDD) which used the coordinates: 4, 50, -4 [11]. We used slightly inferior coordinates for our ROI with the goal of overlapping with a MPFC region shown to have altered functional connectivity to the NAcc in patients with chronic pain which used coordinates: +/-2, 52, -2 [16]. Additionally, we chose more lateralized (x = +/-4) and more posterior (y = 50) coordinates from the previous (MDD patients) study in order to extend lateral coverage of the area, reduce the amount of overlap of the spheres, and to avoid potential prefrontal signal dropout (which is typically found more anteriorly). Our selected ROI was superior and anterior to the MPFC results identified in a previous meta-analysis [17], however, we believe that our selected ROI region was less susceptible to any potential fMRI artifacts within the orbitofrontal cortex (which is a region that often suffers from fMRI data signal drop out due to proximity to the orbital and sinus cavities) [18]. To create our ROI, we first created a MPFC mask image by drawing spheres (AFNI) over a TT_N27+tlrc structural image. Then, we resampled the mask image to match the our fMRI data image dimensions (using AFNI's 3dfractionize, with clip 0.1) which resulted in a mask containing 42 voxels.

FMRI Beta and Questionnaire Variable Correlation Analysis

Arousal (ratings to +$5 cues) was included as a behavioral measure in the correlational analyses because of the group differences identified for arousal (see Fig. 1, whereas valence was not included in the correlational analyses because no group differences were observed). Questionnaire measures included behavioral drive (BIS/BAS, BAS drive subscale) [1], behavioral fun seeking (BIS/BAS, BAS fun seeking subscale), behavioral reward responsiveness (BIS/BAS, BAS reward responsiveness subscale), behavioral inhibition (BIS/BAS, BIS subscale), positive affect (PANAS, PAS subscale) [2], negative affect (PANAS, NAS subscale),

trait anxiety (STAI Trait) [3], state anxiety (STAI State) [3], total mood disturbance (POMS) [4], depression (BDI) [5], pain severity (BPI) [6], pain interference (BPI), fatigue (PROMIS Fatigue) [7], and number of painful body regions (ACR Criteria for Fibromyalgia, Widespread Pain Index Scale) [8].

Correlations between ROI fMRI beta values and questionnaire variables were corrected for multiple comparisons based on the inclusion of 7 independent (not correlated) measures. Several of the questionnaire variables were correlated with each other (using data from the fibromyalgia groups, N=34) and represented 4 independent measures: (1) arousal (not correlated with other measures); (2) behavioral drive and behavioral fun seeking (all $p < 0.001$); (3) behavioral inhibition (not correlated with other measures), (4) behavioral reward responsiveness, positive affect, negative affect, trait anxiety, state anxiety, total mood disturbance, depression, pain severity, pain interference, fatigue, and number of painful body regions (all $p < 0.05$). Only the NVLout ROI fMRI betas were correlated with each other (using data from the fibromyalgia groups, N=34, $r = 0.531$, $p = 0.001$). Therefore, the four ROI fMRI betas represented 3 independent measures: 1) NAcc GVNant, 2) MPFC GVNant, and 3) combined independent measure for NAcc NVLout and MPFC NVLout. Thus, all correlations between ROI fMRI betas and questionnaire variables were Bonferroni corrected for a total of 7 multiple comparisons and considered significant at the level of $p < 0.007$ (corrected threshold).

Analysis for Motion Effects

Motion can be a confounder in any fMRI experiment, and this is especially important to consider when comparing patients to healthy controls because individuals experiencing pain (e.g., chronic pain patients) during scanning may have difficulty remaining still during MRI scans. Because of this we conducted additional post-hoc analyses to confirm that group differences and individual differences in motion during scan acquisition were not confounders of or

contributors to our results. To determine average motion during the MID task scans for each participant, Euclidean norm values (e-norm, calculated square root of the sum of squares across 6 rigid-body motion parameters) were calculated for each fMRI volume. These motion estimates were included as a covariate of no interest in post-hoc analyses (ANCOVA, SPSS) and were found to not change the results. The extracted beta values were statistically analyzed across all three groups using two-tailed bivariate Pearson correlation of ROI fMRI beta values and motion (SPSS). Additionally, all volumes with signal exceeding > 4 standard deviations from the mean activity were removed from raw preprocessed time course data prior to extraction of beta values and statistical analysis of fMRI data [19].

Post-hoc Whole Brain Confirmatory and Supplementary Analysis

A post-hoc whole brain analysis across the 3 groups was conducted to 1) confirm the ROI findings, and 2) potentially inform future research. A whole brain mask excluded all voxels outside of the brain from statistical analysis of the images performed in AFNI (3dANOVA). Resulting F-stat images were thresholded at an uncorrected $p < 0.05$ [(>20 voxels, NN=1 (voxel faces touching)]. Between-group T-stat images were also thresholded at an uncorrected $p < 0.05$ (>20 voxels, NN=1) to show directionality of group differences across the whole brain.

Supplementary Results

Confirmatory Non-significant Relationships between Beta Values and Motion

Post-hoc correlation analyses between motion and ROI fMRI responses revealed no significant relationships: NAcc reward anticipatory response (GVNant betas) x motion ($r = -0.159$, $p = 0.276$, $N = 49$); MPFC reward anticipatory response (GVNant betas) x motion ($r = 0.500$, $p = 0.099$, $N = 49$); MPFC no-loss outcome response (NVLout betas) x motion ($r = -0.153$, $p = 0.292$, $N = 49$).

Confirmatory Post-hoc Whole Brain Analyses

Whole brain fMRI data were compared across the 3 groups for both reward anticipatory (GVNant contrast) and non-loss outcome response (NVLout contrast) and generally confirmed the observed group differences from the ROI analyses (extracted beta values). The overall group differences for the whole brain analyses were consistent with the ROI analyses, but no clusters survived correction for multiple comparisons (results reported here are uncorrected only). Uncorrected F-stat images showed widespread group differences in the MPFC for both GVNant and NVLout contrasts (uncorrected $p < 0.05$). Post-hoc between group whole brain analyses (T-stat images, uncorrected $p < 0.05$) confirmed these overall findings and the ROI results; these images showed regional (e.g., MPFC) differences during gain anticipation and no-loss outcome between non-opioid fibromyalgia and control groups as well as between opioid and non-opioid fibromyalgia groups, but minimal differences between opioid fibromyalgia and control groups (Supplementary Fig. S1 and S2). Additional post-hoc whole brain analyses for the LVNant (loss versus non-loss anticipation) and GVNout (gain versus no gain outcome) contrasts were also conducted to inform future investigations (Supplementary Fig. S3 and S4).

References

1. Carver, C. S. & White, T. L. Behavioral inhibition, behavioral activation, and affective responses to impending reward and punishment: The BIS/BAS scales. *Journal of Personality and Social Psychology.* 319–333 (1994).
2. Watson, D., Clark, L. A. & Tellegen, A. Development and validation of brief measures of positive and negative affect: the PANAS scales. *J. Pers. Soc. Psychol.* **54**, 1063–1070 (1988).
3. Spielberger, C. D., Gorsuch, R. L. & Lushene, R. E. Manual for the state-trait anxiety inventory. (1970).

4. McNair, D. M., Lorr, M. & Droppleman, L. *Manual for the Profile of Mood States*. (Educational and Industrial Testing Service, 1971).

5. Beck, A. T., Steer, R. A. & Carbin, M. G. Psychometric properties of the Beck Depression Inventory: Twenty-five years of evaluation. *Clin. Psychol. Rev.* **8**, 77–100 (1988).

6. Cleeland, C. S. & Ryan, K. M. Pain assessment: global use of the Brief Pain Inventory. *Ann. Acad. Med. Singapore* **23**, 129–138 (1994).

7. Broderick, J. E., DeWitt, E. M., Rothrock, N., Crane, P. K. & Forrest, C. B. Advances in Patient-Reported Outcomes: The NIH PROMIS(®) Measures. *EGEMS (Wash DC)* **1**, 1015 (2013).

8. Wolfe, F. *et al.* The American College of Rheumatology preliminary diagnostic criteria for fibromyalgia and measurement of symptom severity. *Arthritis Care Res.* **62**, 600–610 (2010).

9. Knutson, B., Taylor, J., Kaufman, M., Peterson, R. & Glover, G. Distributed neural representation of expected value. *J. Neurosci.* **25**, 4806–4812 (2005).

10. Wu, C. C., Samanez-Larkin, G. R., Katovich, K. & Knutson, B. Affective traits link to reliable neural markers of incentive anticipation. *Neuroimage* **84**, 279–289 (2014).

11. Knutson, B., Bhanji, J. P., Cooney, R. E., Atlas, L. Y. & Gotlib, I. H. Neural responses to monetary incentives in major depression. *Biol. Psychiatry* **63**, 686–692 (2008).

12. Beck, A. *et al.* Ventral striatal activation during reward anticipation correlates with impulsivity in alcoholics. *Biol. Psychiatry* **66**, 734–742 (2009).

13. Arrondo, G. *et al.* Reduction in ventral striatal activity when anticipating a reward in depression and schizophrenia: a replicated cross-diagnostic finding. *Front. Psychol.* **6**, 1280 (2015).

14. Knutson, B. & Heinz, A. Probing Psychiatric Symptoms with the Monetary Incentive Delay Task. *Biological Psychiatry* **77**, 418–420 (2015).

15. Martucci, K. T., Borg, N., MacNiven, K. H., Knutson, B. & Mackey, S. C. Altered prefrontal

correlates of monetary anticipation and outcome in chronic pain. *Pain* **159**, 1494–1507 (2018).

16. Baliki, M. N. *et al.* Corticostriatal functional connectivity predicts transition to chronic back pain. *Nat. Neurosci.* **15**, 1117–1119 (2012).
17. Knutson, B. & Greer, S. M. Anticipatory affect: neural correlates and consequences for choice. *Philos. Trans. R. Soc. Lond. B Biol. Sci.* **363**, 3771–3786 (2008).
18. Deichmann, R., Gottfried, J. A., Hutton, C. & Turner, R. Optimized EPI for fMRI studies of the orbitofrontal cortex. *Neuroimage* **19**, 430–441 (2003).
19. Sawe, N. & Knutson, B. Neural valuation of environmental resources. *Neuroimage* **122**, 87–95 (2015).

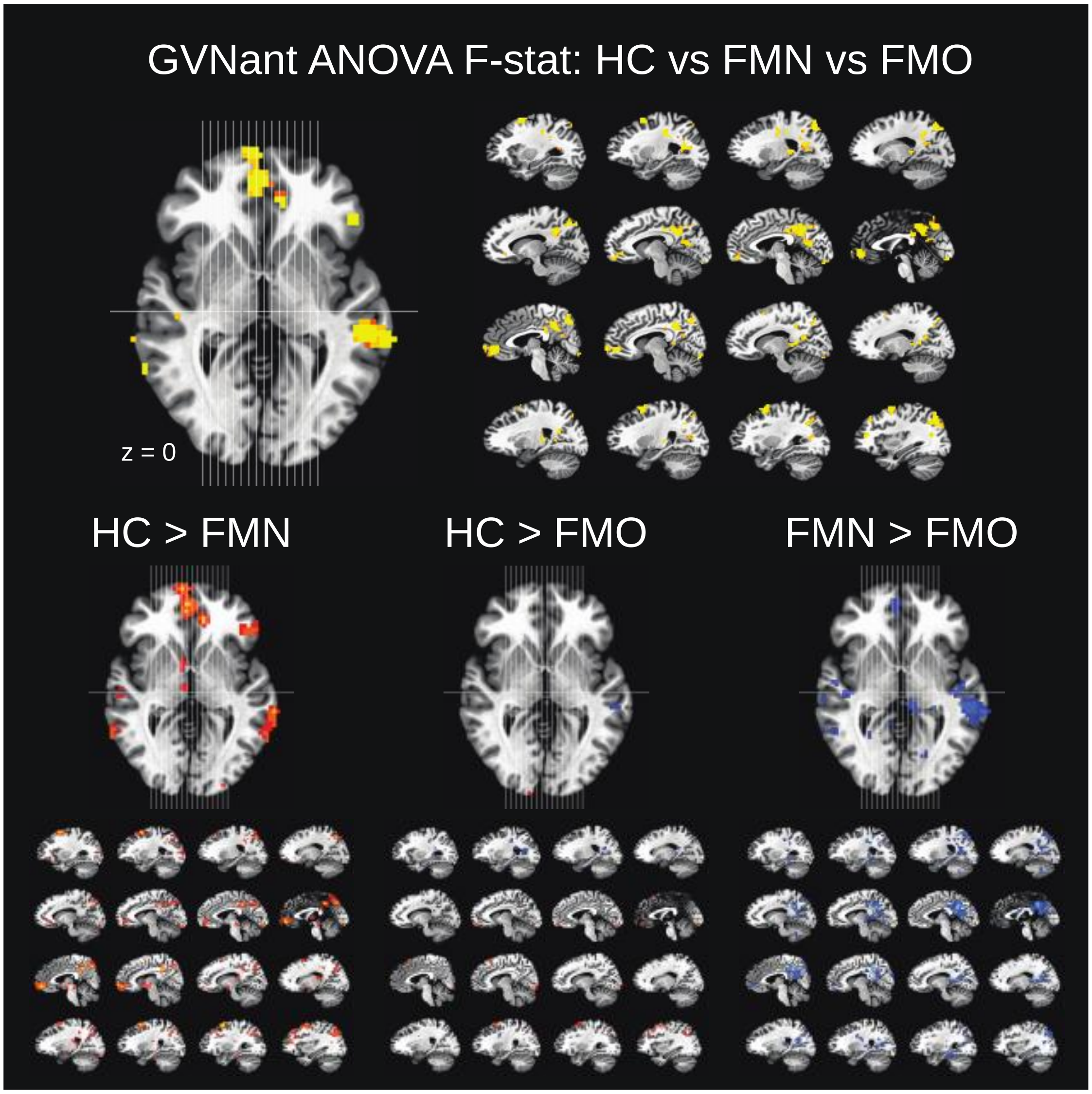

Supplementary Figure S1. Whole Brain Between Group Comparisons for GVNant Contrast. Gain versus no-gain anticipation (GVNant) contrast was analyzed across all three groups for whole brain data. Top panels show uncorrected F-stat results from ANOVA (group effects). Bottom panels show between-group uncorrected T-stat results for each separate group comparison. Warm colors represent increases (red-yellow); cool colors represent decreases (blue-light blue). Healthy controls, HC; fibromyalgia patients not taking opioids, FMN; fibromyalgia patients taking opioids, FMO.

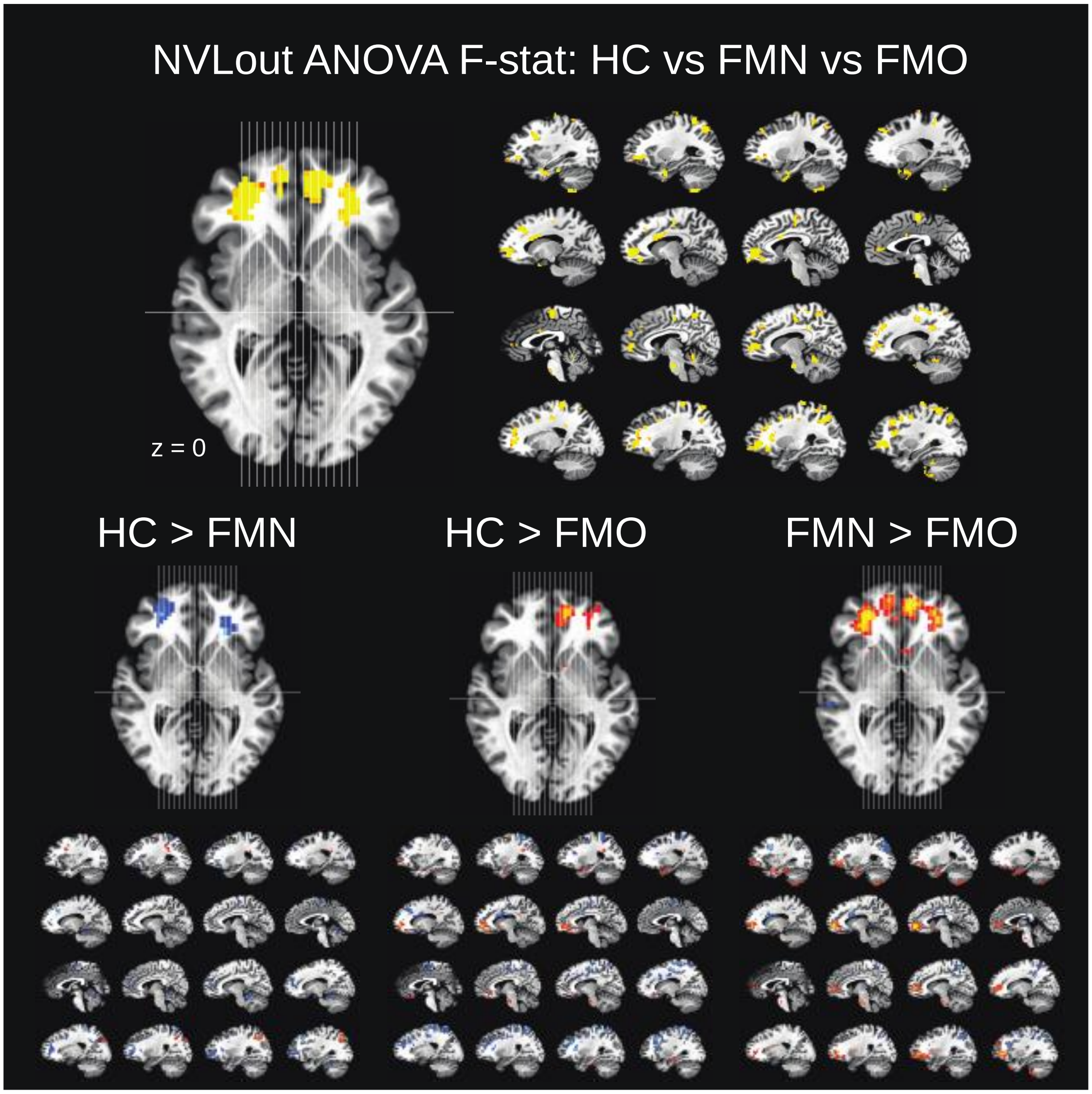

Supplementary Figure S2. Whole Brain Between Group Comparisons for NVLout Contrast. No-loss versus loss outcome (NVLout) contrast was analyzed across all three groups for whole brain data. Top panels show uncorrected F-stat results from ANOVA (group effects). Bottom panels show between-group uncorrected T-stat results for each separate group comparison. Warm colors represent increases (red-yellow); cool colors represent decreases (blue-light blue). Healthy controls, HC; fibromyalgia patients not taking opioids, FMN; fibromyalgia patients taking opioids, FMO.

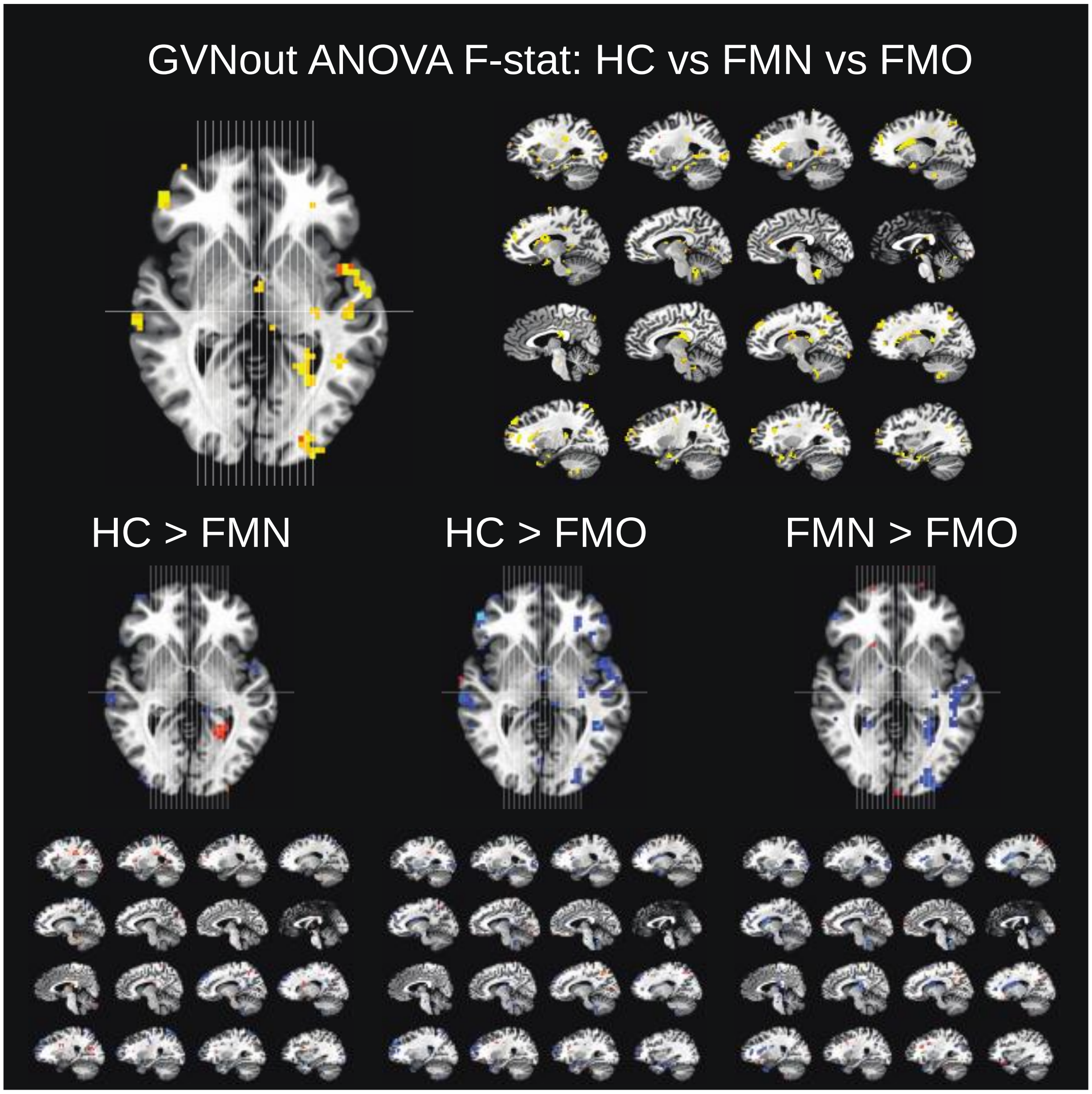

Supplementary Figure S3. Whole Brain Between Group Comparisons for GVNout Contrast. Gain versus no-gain outcome (GVNout) contrast was analyzed across all three groups for whole brain data. Top panels show uncorrected F-stat results from ANOVA (group effects). Bottom panels show between-group uncorrected T-stat results for each separate group comparison. Warm colors represent increases (red-yellow); cool colors represent decreases (blue-light blue). Healthy controls, HC; fibromyalgia patients not taking opioids, FMN; fibromyalgia patients taking opioids, FMO.

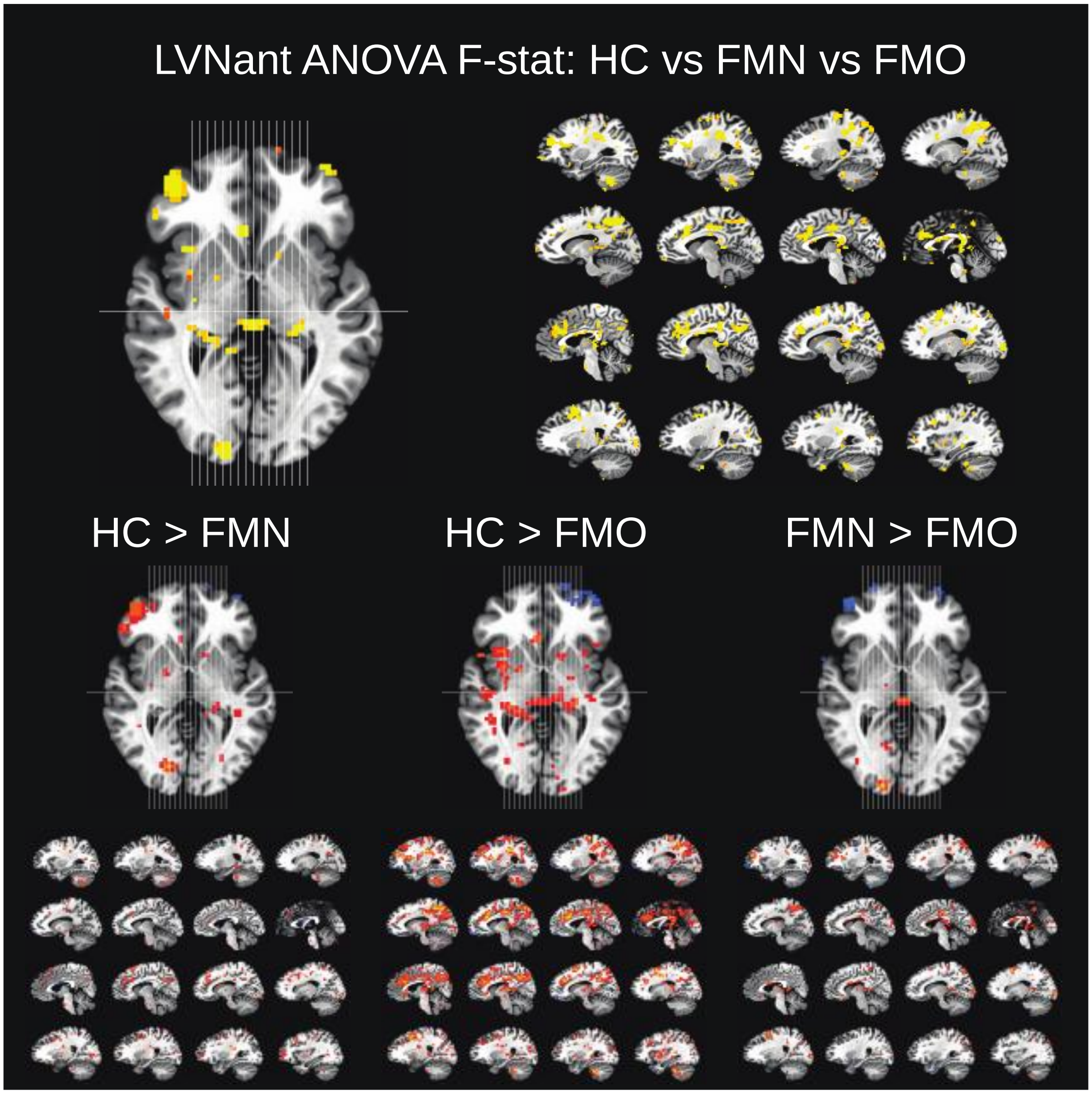

Supplementary Figure S4. Whole Brain Between Group Comparisons for LVNant Contrast. Loss versus no-loss anticipation (LVNant) contrast was analyzed across all three groups for whole brain data. Top panels show uncorrected F-stat results from ANOVA (group effects). Bottom panels show between-group uncorrected T-stat results for each separate group comparison. Warm colors represent increases (red-yellow); cool colors represent decreases (blue-light blue). Healthy controls, HC; fibromyalgia patients not taking opioids, FMN; fibromyalgia patients taking opioids, FMO.

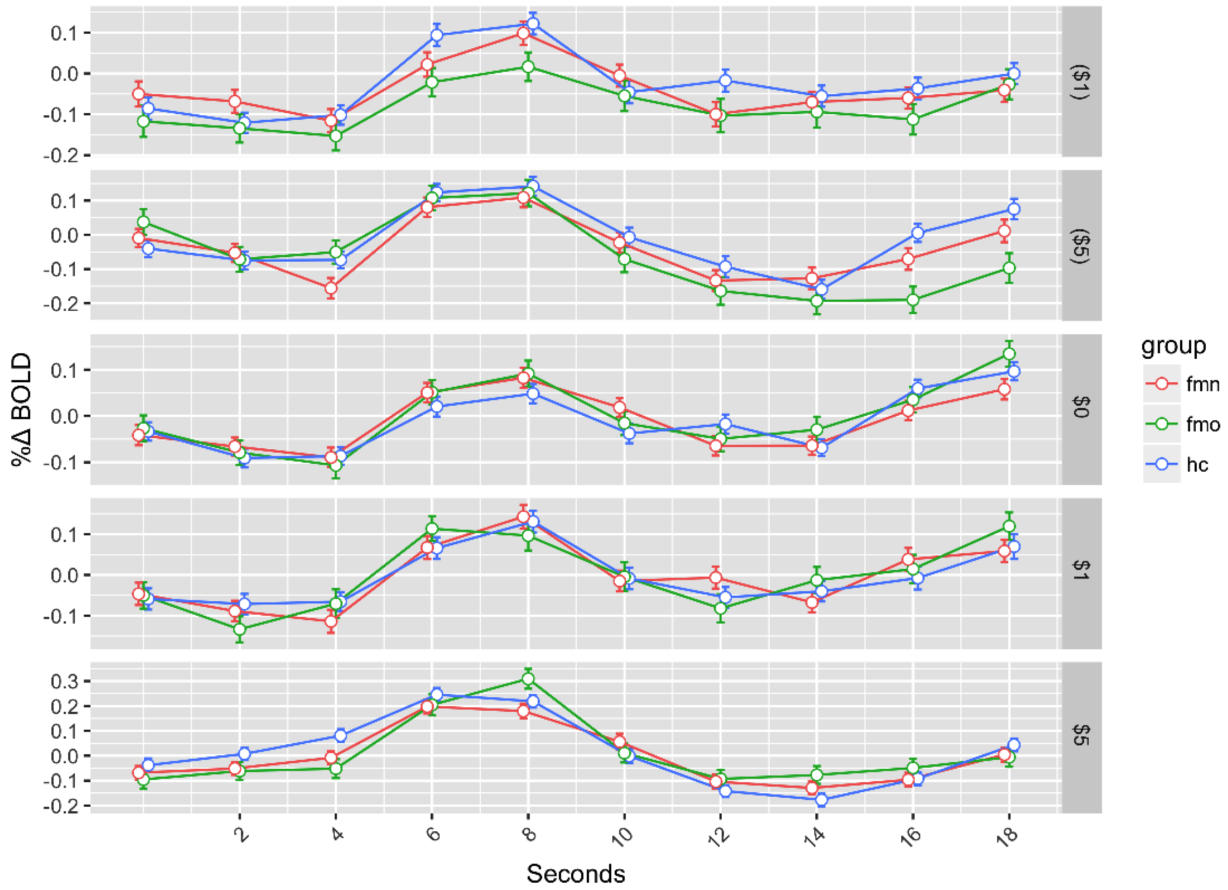

Supplementary Figure S5. Raw Time Course Data for Nucleus Accumbens Across All Cues. Raw time course data (i.e., no fMRI contrast was applied to the preprocessed fMRI data) are shown as % change blood oxygenation level dependent (BOLD) signal. The data were extracted from the nucleus accumbens (NAcc) for all MID Task trials and were plotted separately for each group (colors, see key in figure) and each cue (horizonal panels) and were not chosen based on any specific outcomes (i.e., “hits” or “miss” trial outcomes were disregarded in the data presented here). Cues of negative amounts (e.g., -$1 and -$5) are represented in parentheses in the figure, while cues of positive (potential gain) amounts are not shown in parentheses. The $0 cue represents a combination of -$0 and +$0 cues because the fMRI responses to these cues were essentially indistinguishable. The anticipation fMRI BOLD response was estimated to correspond to 4-8 seconds [cue and fixation during TR 1 and TR 2

(0-4 seconds) plus a 4 second delay accounting for hemodynamic response function (HRF)]. Note the different order of magnitude for +$5 cue trials (bottom panel) as compared with all other trials (in all other panels) (see % change BOLD scales to the left of each panel). Healthy controls, hc; fibromyalgia patients not taking opioids, fmn; fibromyalgia patients taking opioids, fmo.

	NAcc GVNant			NAcc NVLout			MPFC GVNant			MPFC NVLout		
	N	r	p	N	r	p	N	r	p	N	r	p
Arousal Rating	32	-0.139	0.447	32	-0.207	0.256	32	-0.157	0.39	32	-0.145	0.429
Duration of Symptoms (years)	33	-0.004	0.981	33	0.298	0.092	33	-0.043	0.812	33	0.058	0.749
Positive Affect (PANAS)	34	0.06	0.735	34	-0.142	0.424	34	0.181	0.306	34	0.019	0.916
Negative Affect (PANAS)	34	0.088	0.622	34	0.152	0.391	34	-0.024	0.893	34	0.165	0.351
Trait Anxiety (STAI)	34	-0.11	0.535	34	-0.054	0.763	34	-0.271	0.121	34	0.047	0.79
State Anxiety (STAI)	34	0.234	0.182	34	0.048	0.785	34	-0.122	0.494	34	0.127	0.475
Number of Pain Areas (FAF)	34	-0.019	0.917	34	-0.124	0.486	34	-0.084	0.639	34	-0.001	0.996
Fatigue (PROMIS)	33	-0.146	0.416	33	-0.037	0.838	33	0.068	0.709	33	-0.169	0.346
Behavioral Drive (BAS)	30	0.165	0.384	30	0.225	0.232	30	0.05	0.794	30	0.507**	0.004
Behavioral Fun (BAS)	30	0.157	0.407	30	0.155	0.412	30	0.184	0.33	30	0.365*	0.047
Behavioral Reward (BAS)	30	-0.101	0.596	30	-0.082	0.667	30	0.397*	0.03	30	-0.005	0.979
Behavioral Inhibition (BIS)	30	0.059	0.757	30	-0.118	0.535	30	0.072	0.705	30	0.047	0.805
Depression (BDI)	34	0.06	0.734	34	0.111	0.533	34	0.12	0.498	34	-0.049	0.783
Mood Disturbance (POMS)	34	0.18	0.308	34	0.084	0.637	34	-0.338	0.051	34	0.166	0.349
Pain Severity (BPI)	34	0.084	0.639	34	0.197	0.263	34	-0.1	0.574	34	0.082	0.646
Pain Interference (BPI)	34	-0.138	0.436	34	-0.027	0.88	34	-0.176	0.319	34	-0.179	0.312

Supplementary Table S1. Correlations between ROI Betas and Questionnaire Measures. Participant counts for each measure differ from the total number of participants (controls N=15, non-opioid fibromyalgia (FM) N=17, opioid FM N=17) because some participants did not complete all questionnaires. Abbreviations: NAcc, nucleus accumbens; MPFC, medial prefrontal cortex; GVNant, gain versus no-gain anticipation contrast; NVLout, no-loss versus loss outcome contrast; PANAS, Positive and Negative Affect Schedule; BIS/BAS, Behavioral Inhibition System/Behavioral Activation System; PROMIS, Patient-Reported Outcomes Measurement Information System; STAI, State-Trait Anxiety Inventory; FAF, Fibromyalgia Assessment Form; BDI, Beck Depression Inventory; POMS, Profile of Mood States; BPI, Brief Pain Inventory. Data are presented for descriptive purposes only, therefore Pearson correlation (r) and significance values (p, P-Value) shown are not corrected for multiple comparisons. * $p < 0.05$, ** $p < 0.01$
